# Don’t Go Chasing Narcissists: A Relational-Based and Multiverse Perspective on Leader Narcissism and Follower Engagement Using a Machine Learning Approach

**DOI:** 10.1177/01461672221094976

**Published:** 2022-05-27

**Authors:** Dritjon Gruda, Dimitra Karanatsiou, Paul Hanges, Jennifer Golbeck, Athena Vakali

**Affiliations:** 1 Maynooth University, Ireland; 2 Aristotle University of Thessaloniki, Greece; 3 University of Maryland, College Park, USA

**Keywords:** narcissism, leader, follower, personality, machine learning, multiverse analysis

## Abstract

Although research interest in leader narcissism has been on the rise over the past few years, prior literature has predominantly discussed leader narcissism from a leader-centric perspective. In this article, we provide a relational-based perspective of leader narcissism by examining the interaction between follower personality traits and leader narcissism on follower engagement in an online context. We combine a machine learning (ML) approach and multiverse analysis to predict the personality traits of a large sample of leaders and engaged followers across 18 created multiverses and analyze hypothesized interactions using multilevel regressions, also accounting for leader gender moderation effects. We find that the interaction between leader narcissism and follower agreeableness and follower neuroticism positively predicts follower engagement, whereas the interaction between leader narcissism and follower openness negatively predicts follower engagement. In addition, we find that leader gender plays an important moderating role. Limitations and implications are discussed.

## Introduction

Starting with the work by Conger (1990), leadership scholars have been increasingly focused on the destructive or negative side of leadership (e.g., Galvin et al., 2010; Harms et al., 2011; Judge et al., 2009). People who score high on these so-called dark triad traits (narcissism, Machiavellianism, and psychopathy) tend to enact destructive styles of leadership. Although three dark traits have been identified, scholars have tended to concentrate on narcissism when studying destructive leadership (e.g., Maccoby, 2004; Rauthmann & Kolar, 2013). Narcissists are people whose decisions and goals are driven by unrelenting arrogance and self-absorption (Rosenthal & Pittinsky, 2006). Such individuals lack empathy, have fragile self-esteem, and are hostile to others who threaten their positive self-regard (Rosenthal & Pittinsky, 2006).

To date, the majority of the published studies have used a leader-centric perspective for understanding the consequences of leader narcissism. A leader-centric perspective means that researchers only examine the leader’s characteristics and attributes to understand the consequences of narcissism (Northouse, 2019). For example, researchers have examined whether leader personality traits, such as extraversion or neuroticism (based on Miller et al., 2018), interact with leader narcissism to determine the consequences of this dark trait.

Unfortunately, using a leader-centric perspective only provides an incomplete image of leadership. In contrast to this perspective, researchers are increasingly emphasizing the role of followers in the leadership process (Northouse, 2019; Padilla et al., 2007; Smith et al., 2018). Indeed, in their discussion of the toxic triangle, Padilla et al. (2007) argue that destructive leadership will occur when the right kind of leaders are matched with the right kind of followers in the right context. When followers are given their rightful attention, leadership switches from a focus on individual-level characteristics of a leader to a dynamic and emergent phenomenon that operates at a dyadic or group level of analysis. In the current study, we take a relational-based perspective to understand the consequences of narcissistic leadership. This perspective emphasizes that the leadership process is the joint, co-creation between leaders and followers (Uhl-Bien et al., 2014). We focus on the traits of both leaders and followers to understand the consequences of leader narcissism. We maintain that follower attraction to certain leaders as well as the success of followers engaging with these leaders is a function of both leader and follower traits (Gruda, Karanatsiou, et al., 2021). When followers’ traits are mismatched with the leader, followers either transfer to other groups or eventually become part of the leaders’ out-group (Decoster et al., 2013). On one hand, we believe that putting followers back in the leadership equation adds to the literature on destructive leadership. We argue that certain types of leaders and followers combine to form destructive leadership if the environment (in this case social media) is conducive. In this article, we attempt to identify the exact set of followers’ personality characteristics most susceptible to the influence of such leaders, namely, narcissistic leaders. On the other hand, this article also clarifies some of the inconsistent findings concerning narcissistic leadership. For example, studies have found that narcissistic leadership can result in positive and negative consequences (for a review, see Smith et al., 2018). The relational-based perspective answers recent calls for incorporating followers into this literature and has important implications for managers, executives, and organizations.

Another contribution of this article is its setting, namely, the study of leader–follower interactions on social media. Traditional leadership research is commonly based on leaders (i.e., those who have and use their control of resources and organizational authority to influence others) and followers (i.e., those who follow leaders because of the degree of control of resources leaders within the same organization can exert upon them) within clearly defined social groups (i.e., organizations). However, much less is known about the reach of leadership beyond organizational boundaries through using tools such as social media. In the present work, we study the interactions between leaders (i.e., those who influence others, not just organizational followers but also the public, based on their communications online) and followers (i.e., those who follow and interact with leaders not because they have to, but because they choose to follow them) beyond traditional organizational boundaries. Therefore, in addition to providing a relational view of leadership, the present work is a portrayal of how leaders can use social media to expand their reach beyond organizational boundaries and influence the public at large (Matthews et al., 2021). As such, the power that leaders can wield on social media in influencing those that lie beyond organizational boundaries (i.e., the public) must not be discounted.

In sum, the present study takes a relational-based perspective to explore the leader narcissism–follower outcome relationship. Specifically, we test the moderating role of follower personality on the relationship between leader narcissism and follower engagement. We also examined leader gender to understand whether our obtained results operate differently for males and females. Our study is unique in that we tested our hypotheses in informal and leader–follower relationships on an online micro-blog platform (i.e., Twitter) using machine learning (ML) prediction models (Gruda et al., 2020) to predict narcissism and Big Five personality traits. The information systems literature has found that text statements can be used to accurately predict the personality traits of individuals (Gruda & Hasan, 2018, 2019; Gruda & Ojo, 2021, 2022; Gruda, Ojo, et al., 2021; Karanatsiou et al., 2020). Using the ML approach to estimate individual characteristics allowed us to test our hypotheses in a large sample of leaders and followers, including a multiverse analysis of the available data.^1^ However, before discussing our specific hypotheses and the ML algorithm, we provide an overview of existing narcissism and leadership literature.

## Narcissistic Leadership—A Relational Perspective

Personality traits are dispositions that represent individuals’ tendencies to think, feel, and behave in a particular manner, and individuals who score high on the personality trait narcissism are often referred to as narcissists (Judge et al., 2009). Narcissistic individuals often engage in self-enhancing and self-promoting behavior, which can result in initially being perceived as charming (Spain et al., 2014). However, these positive attributions tend to be short-lived, and long-term interactions with such individuals usually result in describing such individuals as untrustworthy and uncaring about others (Grijalva & Newman, 2015). These changed perceptions over time are due to narcissists being driven by their needs for power and admiration and being less concerned with the concerns of others or their organizations (Rosenthal & Pittinsky, 2006).^2^

The existing literature on narcissistic leadership has adopted a leader-centric perspective. This literature has documented that narcissistic leaders are overly concerned with their own reputation and interpret information in a biased and self-serving fashion. Such leaders manipulate conservations toward their own “interests and accomplishments, and arrogantly fantasizing grandiose dreams” (Judge et al., 2009). Consistent with evolutionary psychologists, narcissists tend to be identified as leaders by others. These positive consequences for narcissists have been documented in business organizations and at the national level. For example, Deluga (1997) found a positive relationship between narcissistic entitlement, self-sufficiency, and perceptions of the charisma of U.S. Presidents. Also, narcissists more often emerge as leaders in social contexts requiring agentic behavior (Petrenko et al., 2016) and in highly visible, more public tasks (Wallace & Baumeister, 2002). Hence, narcissistic traits seem to be beneficial to leaders in certain contexts.

A few studies have examined whether other leader characteristics (e.g., personality) might moderate the effectiveness of narcissistic leaders. For example, empirical findings suggest that narcissism is positively correlated with leader extraversion (Grijalva, Harms, et al., 2015) and negatively correlated with leader agreeableness and conscientiousness (Douglas et al., 2012). Unfortunately, prior research has focused on leader traits and contexts. The follower’s role in narcissistic leadership has been ignored to date. As noted in their article on the toxic triangle, Padilla et al. (2007) argued that destructive leadership occurs when a particular set of leadership characteristics attract a particular kind of followers in a conducive or “toxic” environment. Our relational-based perspective suggests that follower characteristics are important for the success of these narcissistic leaders. The traits of some followers might make them susceptible and attracted to such leaders, and others less so. In the next section, we examine the Big Five personality characteristics and hypothesize how these personality factors for followers could moderate the leader narcissism–follower engagement relationship.

### Leader Narcissism and Follower Personality

#### Follower openness

Individuals who score high on openness to experience tend to be curious, visionary, and imaginative (Costa & McCrae, 1992). Given that narcissistic leaders usually exhibit great enthusiasm and express enticing visions of the future, it may appear at first that such followers would be drawn to narcissistic leaders. However, the literature shows that followers who score high on openness frequently have many interests and thus are likely to interact with many others (McCrae & Costa, 1987). This attraction to and connection with multiple others allows followers to easily switch between leaders and possibly even interact with several leaders at once. Previous studies confirm that followers who are open to experience often express less commitment to their organization and teams (Erdheim et al., 2006). Hence, followers who score high on openness to experience might be less susceptible to narcissistic leaders than their counterparts who score low on openness to experience:

**Hypothesis 1 (H1):** The leader narcissism–follower engagement relationship will be moderated by follower openness such that the magnitude of this relationship will be negatively related to followers’ openness to experience.

#### Follower agreeableness

Agreeable individuals tend to be trusting, cooperative, modest, compliant, and tender-minded (Costa & McCrae, 1992). Highly agreeable followers are likely to avoid interpersonal conflict with others, experience higher levels of stress when there is a conflict (Suls et al., 1998), and can come across as “too nice” to others (Ng et al., 2005). These followers may be attracted to and influenced by narcissistic leaders because such leaders appear to be powerful and confident enough to handle future interpersonal conflicts (Smith et al., 2018). We, therefore, predict that agreeable followers are more likely to be drawn to and engaged with narcissistic leaders. We expect the attraction and engagement with narcissistic leaders will diminish as agreeableness decreases:

**Hypothesis 2 (H2):** The leader narcissism–follower engagement relationship will be moderated by follower agreeableness such that the magnitude of this relationship will be positively related to follower agreeableness.

#### Follower neuroticism

Neurotic individuals experience a high degree of anxiety and worry about the future and are more prone to impulsivity and vulnerability. However, as the majority of previous studies have focused almost exclusively on leader neuroticism (Kaiser et al., 2015), little is known about how follower neuroticism interacts with leader narcissism. One exception is a recent study by Gruda, Karanatsiou, et al. (2021) who found that narcissistic leaders seem to attract highly trait-anxious followers. They argue that trait-anxious individuals tend to be more vigilant toward potential threats and therefore tend to worry more than individuals who score low on trait anxiety. Given that anxiety and neuroticism are closely related (Gruda & Hasan, 2019), we hypothesize that neurotic followers likely are drawn to narcissistic leaders because such leaders take control (Petrenko et al., 2016) and exhibit confidence (Gruda, Karanatsiou, et al., 2021), which reduces perceived environmental uncertainty and chaos (Smith et al., 2018):

**Hypothesis 3 (H3):** The leader narcissism–follower engagement relationship will be moderated by follower neuroticism such that the magnitude of this relationship will be positively related to follower neuroticism.

#### Follower extraversion and conscientiousness

Extraverts are assertive, excitement-seeking, and like to engage in social activities (Costa & McCrae, 1992). Not surprisingly, extraverts are more effective and exhibit greater confidence in social interactions (Judge et al., 2002). Unfortunately, the lack of research on extraversion in followers makes it unclear whether such individuals would be more or less likely to engage with narcissistic leaders.

Conscientious individuals are achievement-oriented, disciplined, responsible, and prefer orderliness (Costa & McCrae, 1992). Such individuals tend to be less adaptable to change and might also be obsessive-compulsive (Carter et al., 2016). In traditional organizational settings, highly conscientious followers are likely to follow the instructions of their superiors and less likely to engage in uncivil behavior (Taylor & Pattie, 2014). Yet, it is questionable whether highly conscientious followers are more or less likely to engage in interactions with narcissistic leaders, precisely because of their affinity to follow rules and order. Due to the lack of existing research on follower extraversion and conscientiousness, hypotheses regarding these two follower personality traits acting as moderators of the leader narcissism–follower engagement relationship are more ambiguous. Thus, while we examine these possible interactions, we do so solely in an exploratory manner.

#### Follower narcissism

The Big Five personality traits are not the only follower characteristics that can interact with leader narcissism to affect follower engagement. Followers can be narcissistic just like their leaders (Post, 1986; Rosenthal & Pittinsky, 2006). The attraction similarity hypothesis predicts that similar people initially are more strongly attracted to one another and their relationships are more positive and long-lasting. Empirical research has supported this hypothesis (Morry, 2005, 2007). Hence, it seems that at least initially, narcissistic followers seek out similar leaders in an attempt to find, relate to, and admire others for their status, “power, beauty, intelligence or moral stature” (Post, 1986, p. 679). However, the long-term moderating effect of follower narcissism on the leader narcissism–follower engagement relationship has not been explored. We expect that over time any positive effects of narcissistic leaders will be short-lived with narcissistic followers as well. Therefore, we hypothesized,

**Hypothesis 4 (H4):** The leader narcissism–follower engagement relationship will be moderated by follower narcissism such that the magnitude of this relationship will be negatively related to follower narcissism.

### The Moderating Role of Leader Gender

Followers perceive female leaders differently from male leaders (Thoroughgood et al., 2011). Due to perceptual biases and the consequences of organizational glass-ceiling/glass-labyrinth (Eagly et al., 2007; Hymowitz & Schellhardt, 1986), females have to overcome more obstacles and challenges than males to be perceived as leaders (Ingersoll et al., 2017). From the leader’s perspective, when a female breaks through these barriers and becomes a successful leader, other barriers arise such as potential backlash from peers (Thoroughgood et al., 2011). Put differently, to be successful, female leaders must integrate their identity as a female with their identity as a leader. The extent to which one’s gender and leader identities are perceived as compatible with each other is referred to as “gender-leader identity integration” (Chen, 2018; p. 339). Leaders are expected to behave both agentically and determinedly (Capezio et al., 2017). From the follower’s perspective, expectations and positive attributions for agentic behavior are not extended to female leaders (Keck, 2019). While males can be considered a leader if they are only agentic, females must display both gender-based communal and leader-based agentic behavior to be accepted by their followers (Ayman & Korabik, 2010; Keck, 2019).

We argue that narcissism might be beneficial to both females and males in terms of leader emergence (Grijalva, Newman, et al., 2015). However, beneficial effects probably occur through different mechanisms. Male narcissists likely emerge as leaders due to their tendencies for self-promotion and high confidence (Rosenthal & Pittinsky, 2006), whereas female narcissists emerge as strong leaders due to their disregard for societal gender expectations (Chen, 2018; Thoroughgood et al., 2011). Although female narcissists benefit from their disregard for gender expectations, male narcissists are more likely to be perceived as stronger leaders because of the congruence of stereotypic male behavior and expected agentic behavior in leaders:

**Hypothesis 5 (H5):** The leader narcissism–follower engagement relationship will be moderated by leader gender such that the magnitude of this relationship will be larger for males than for females.

Although not explicitly hypothesized, we also tested whether leader gender moderated any of the previously hypothesized moderation of the leader narcissism–follower personality interactions. We do not make specific expectations about the direction of these three-way interactions; rather, we treated these analyses as exploratory. In general, however, we expect male narcissistic leaders to benefit from the positive tendencies of their narcissistic disposition while expecting that this relationship will be less evident in the case of female narcissistic leaders.

## Context: Online Environment

As indicated earlier, destructive leadership emerges when there is a match between leader and follower traits in an environment conducive to unethical behavior. We expect that the attraction between followers and leaders is stronger in uncertain and chaotic contexts. In the current study, we study the leader–follower process in the online social media environment. The social media environment can be described as dynamic (i.e., updates continuously throughout the day), uncertain (e.g., personal attacks can be made against the original poster), and chaotic (e.g., the chain of logic might deteriorate into name-calling or a meaningful exchange of ideas). In other words, the social media environment is conducive to destructive leadership.

In the present study, we examine data derived from the social media platform Twitter. Twitter has more than 330 million monthly active users (statista.com) and is the world’s most popular microblogging platform. On average 6,000 tweets (up to 280-character text messages) per second or 500 million tweets per day are posted on the platform. Twitter users also can subscribe to other users’ posts, who are known as followers. Twitter users’ news feed captures a user’s thoughts, feelings, and conversations at any moment in time as microblogs are quick, short, and mostly capture what is going on at any particular moment in users’ lives (Gruda & Hasan, 2019).

We recognize that interactions on Twitter between individuals, namely, “leaders” and their followers, may initially appear to be conceptually different than more traditional interactions between organizational leaders and their followers. We contend that when social media leadership is examined in more detail, the generalizability of social media leadership to more traditional settings becomes apparent for multiple reasons.

First, we define leaders as individuals who are followed by others on Twitter. Just like informal leaders in traditional work environments, a regular employee (i.e., someone without managerial responsibilities and team oversight in their usual workplace) oftentimes can be perceived as a leader on social media platforms such as Twitter. Indeed, one could argue that similar to more traditional organizational leaders, Twitter leaders try to influence followers’ communication content and patterns and can yield large referent and expert power. For example, Twitter leaders who are an expert in some field (e.g., a scientist, technician, craftsman, or journalist) and/or whose followers can identify with, often have a greater number of loyal online followers than those not considered an expert or likable (Ghosh et al., 2013). This might be the case, albeit these same leaders might not have managerial responsibility and supervisory oversight of others at their actual workplace. And similar to more traditional leaders, Twitter leaders are trying to influence followers’ communication content and patterns.

Second, the bases of power (French, 1962) used by traditional leaders and social media leaders are substantially the same. Although there is no formal authority structure that can be used to influence followers in the social media world, Twitter leaders can use both expert and referent power to influence their followers. Furthermore, these Twitter leaders can also use reward and coercive power. Twitter leaders can reward a follower by retweeting their comments or the leader can use coercive tactics by ridiculing or posting harsh criticism of the follower. Thus, although there is a lack of a formal structure providing legitimate power, social media leaders do indeed use many of the same bases of power as other more traditional organizational leaders (Gruda, Karanatsiou, et al., 2021).

Third, the traditional literature repeatedly highlights that leaders influence their followers in several ways (Hanges et al., in press). Previous literature highlights the central role of leader communication and that literature views communication as a critical and fundamental component of leadership (Fairhurst & Connaughton, 2014). Communication quality, quantity, and content (e.g., vision statements) are positively related to perceived leadership performance (Neufeld et al., 2010), overall team performance (Marks et al., 2000), and the effectiveness of transformational and charismatic leaders (Berson & Avolio, 2004). Leader communication provides a frame of reference (Bateson, 1972; Goffman, 1974) due to activated schemas that facilitate followers’ sense-making of their environment (e.g., Fairhurst & Connaughton, 2014). Although essential for face-to-face teams, the importance of leaders’ communication effectiveness is enhanced in a virtual context (Berson & Avolio, 2004). And with the sudden and discontinuous changes to the work environment caused by the COVID-19 pandemic (McCleskey & Gruda, 2020) highlighting the importance of leader–follower research in virtual environments, examining leadership in a social media context may provide the perfect laboratory for understanding this work in the future.

Finally, studying leader–follower relationships in an online context removes several possible exogenous factors, such as switching costs, which might play a role in shaping followers’ perceptions of their leaders. Prior research oftentimes equates followership with subordinates of a specific organizational leader. Various macro-economic factors, such as the state of the economy, industry growth, competition circumstances, can make it difficult for employees to switch leaders or organizations. Thus, employees can get “stuck” in undesirable employer–employee dynamics. These macro-economic factors and individual-level factors (e.g., work experience and career growth) are contaminating factors that distort findings regarding the consequences of leader and follower characteristics. In online contexts, none of these factors play a role as switching costs for followers are virtually nonexistent. If followers do not agree with one leader or do not derive any benefit from following that person, they can easily “unfollow” their leader.

## Method

Our sample is based on a data set of organizational employees and their company information provided by Crunchbase (crunchbase.com). Crunchbase is a platform that provides business information about private and public companies. Among data sets that include investments and funding, Crunchbase also provides a list of founding members, senior leaders, and organizational employees for each listed company. Many of the provided people profiles also include links to social media accounts, including Twitter. Our original data set, after excluding all non-U.S. companies and dropping profiles for which Twitter links were not available, consisted of 60,872 individuals and their full social media URLs and business information (e.g., company and job title). However, not all of these individuals are suitable for analysis given the parameters of this study. Hence, to avoid selection bias and ensure our examined sample was feasible for analysis, we implemented several selection criteria for both leaders and followers:

- *Specific timeframe to measure engagement*: To ensure that our analyses would not be affected by censored data, we retained individuals whose tweets were published within an examined time frame of January 1, 2018, to November 15, 2019. Given the pace of activity on Twitter, we reasoned that a sufficient amount of time had passed so that our dependent variable, follower engagement, would not be biased at the end date of our data collection efforts.
- *Unique engagement measurement/inclusion in original posts*: We removed any retweets to prevent double-counting of follower responses.
- *Followers engaged in leaders’ post identification*: We gathered all the tweets from users that engaged with the leader within the aforementioned time period of this study. Follower engagement was defined as the number of *unique followers’ replies to a leader’s tweet*. To ensure that our follower engagement dependent variable was not overrepresented by relatively inactive followers, we excluded followers (and their respective leaders) who had engaged with the leader fewer than 4 times. We counted the number of follower replies, rather than follower likes or retweets of leader posts because replying to a tweet requires an expenditure of effort and provides information about the nature of the follower’s communication intention (Ferrara et al., 2016). This led to a preliminary data set of 917 leaders and 70,523 followers. Throughout the remainder of this article, we refer to this data set as our base data set.
- *Spam removal and behavioral criteria refinements*: Using the base data set, we applied various metrics to lower the likelihood of bot-like accounts in our data set (for an overview, see Cresci et al., 2015). We selected only those leaders who posted a minimum of 100 tweets and who had at least 30 unique followers within our data set. Doing so reduced the leader count to 914 leaders. The minimum criterion of 100 tweets with 30 follower observations allowed us to identify leaders with sufficient data to meaningfully test our hypotheses. As leaders in our data set were derived from a prescreened database, we were confident that all included leaders were real users and not bots. However, to ensure we had not accidentally included bots (or bot-like accounts) in our examined follower sample, we also only included followers in our final data set who had posted a minimum of 100 tweets (i.e., 69,490 followers), had at least one friend (i.e., two users follow each other; 69,311 followers), had at least 30 followers themselves (i.e., 65,545 followers), and were included on at least one Twitter list (58,877 followers):

Twitter Lists display tweets from a curated list of Twitter accounts, allowing users to customize, organize and prioritize the Tweets they see on their timeline. Twitter Lists can be shared, subscribed to and created based on topics of interests, specific events or groups of people (e.g., inspiring leaders).

Based on our definition of leaders as “individuals who are followed by others on Twitter,” we considered leadership to be an influence over others, regardless of the status and authority or power the leader holds over their followers. Having said that, most leaders in our data set (e.g., Model 1) do indeed hold traditional leadership positions in organizations. For example, approx. 41.19% of selected leaders in our data set are executive leaders (CEO, CFO, Founder, etc.), 11.96% are senior leaders (Managing Directors, Executive Vice Presidents, Senior Directors, etc.), and 11.27% hold a managerial position (Senior Manager, Vice Presidents, Marketing Manager, etc.). Therefore, in total, 64.42% of selected leaders in our data set indeed hold leadership positions within their respective organizations. As such, this data set largely (but not solely, see our definition of leaders in this article) constitutes organizational leaders who are using their social media to reach beyond organizational boundaries and influence others.

With regard to our dependent variable, while we agree that in principle looking at alternative metrics such as follower engagement is reasonable, we decided not to rely on variables such as retweets or likes, but rather chose to focus on follower engagement (i.e., the number of follower replies to a leader’s tweet) for several reasons. First, alternative metrics such as retweets and likes are easily manipulatable by bots (Ferrara et al., 2016). Therefore, such measures oftentimes can provide a false impression of engagement. Hence, using such metrics would not likely provide valid results.

Second, assuming such metrics of a leader are solely provided by human followers (not bots), both retweets and likes constitute low-effort variables. Hence, for a follower to read a leader’s tweet and hit the like button hardly constitutes a form of engagement given the ease and speed with which likes or retweets can be conducted. And because such metrics do not require reflection on the followers’ part, we would hesitate to use such metrics as an equivalent to follower engagement. On the contrary, commenting on a leader’s tweet, even when indicating disagreement with the content of the leader’s tweet or the leader themselves, constitutes a form of effort and some degree of reflection. Hence, we argue that followers’ replies to leaders’ tweets are a high effort variable and minimize the noise in our models when compared with other metrics.

Third, the growing literature on followership has recognized that not all followers are the same. Indeed, several typologies have been proposed (Chaleff, 2009; Kelley, 1988) to account for different types of followers. Of these different typologies, Kelley’s (1988, 2008) framework is the most widely cited and used. Kelley identifies five different types of followers that can be differentiated by two underlying dimensions (active/passive; independent, critical thought/dependent, uncritical). The two types of followers who are more active are Conformist (those who are engaged followers who do what the leader wants) and Exemplary (i.e., followers who are engaged and in-group but will raise issues and argue with the leader if they disagree). Note that Kelley labels such followers “Exemplary,” which describes followers who are not afraid to call out the leader when necessary. Follower engagement reflects all types of followers and hence does not limit the scope of a leader’s influence to one specific audience (or type of followers).

It should be noted that we considered followers to be any Twitter users who interacted with the selected leaders, regardless of whether these users actually “followed” the leader’s Twitter account. For example, a CEO’s followers were solely determined based on which users interacted with the CEO on Twitter, regardless of the follower status or whether they belonged to the same organization as the leader. Hence, not all users who engaged with a leader on Twitter were necessarily followers of that leader on Twitter. A Twitter user might, for example, come across a post by a leader because the respective post has “gone viral.” Yet, most of the time, an engaged user (i.e., a user who interacts with a leader on Twitter) would also be a Twitter follower of that leader. Indeed, based on a random subset of our base sample, we found that 78.3% of engaged users in our data set were also Twitter followers of a particular leader account. In other words, most of our users were actively engaged with their leaders’ tweets and therefore could accurately be considered followers and not bots or random users. To this data set of leaders and their corresponding followers, we applied pretrained ML models to annotate accounts with narcissism and personality scores.

### Multiverse Analysis and Data Preprocessing

We recognized that the decisions we had to make to clean and preprocess our base data set might affect our results. There was no clear answer with regard to the right cut-offs to use and that introduced the possibility of researcher degrees of freedom as well as a challenge to selecting one path from a series of plausible steps. To provide transparency regarding the consequences of our preprocessing decisions on our results, we conducted and presented a multiverse analysis, as suggested by Steegen et al. (2016). A multiverse analysis refers to creating a new data set for each possible combination of plausible processing steps and rerunning our multilevel model for each sub-dataset. Conducting a multiverse analysis allowed us to determine and document which results are robust across preprocessing options (Steegen et al., 2016). In our case, the respective preprocessing choices included the (a) number of interactions between leaders and followers (i.e., number of leader–follower engagements per leader), (b) inclusion and exclusion of more popular leaders from our data set, and, finally, (c) ratio between friends count and follower count (friend count/follower count^2^; Cresci et al., 2015). We provide more information regarding these options below:

- *The number of leader–follower interactions*: As specified earlier, the minimum number of follower engagements with their leaders was set at four, to ensure that our follower engagement dependent variable was not overrepresented by relatively inactive followers. In addition, to make broader generalizations and conclusions, we also investigated whether results remained robust in data sets with a higher minimum number of interactions. Previous research has shown that, initially, narcissistic leaders can come across as charismatic, confident, and even likable (Grijalva, Harms, et al., 2015). However, over time, as followers get to know their narcissistic leaders better, such leaders no longer are seen as positively as they once were. Hence, it is likely that the number of interactions between leaders and followers can influence the robustness of our expected results, as outlined in our hypotheses. Therefore, in our multiverse analysis, we examined results based on a minimum number of four, five, and six leader–follower interactions. The number of chosen leader–follower interactions (i.e., 4, 5, and 6) was primarily due to data limitations. For example, while our data set includes a minimum of four interactions (i.e., baseline), focusing on five interactions results in a drop of 33.61% of available data in our data set, and focusing on six interactions results in a drop of 48.89% of available data. Specifying a higher minimum, for example, 10 would result in a drop of over 75.88% of available data overall. Given the large associated drop in data to facilitate such analyses, we decided against this step. Instead, we show that the outlined number of interactions yields useful and interpretable insights into the role of leader and follower personality traits when the number of leader–follower interactions increases.
- *Inclusion and exclusion of more popular leaders*: Another factor that might influence our results is the popularity of the leaders included in our database. Recent research examining leader–follower interactions on social media, for example, found that leader popularity changed the expected effects that narcissistic leaders have on highly anxious followers (Gruda, Karanatsiou, et al., 2021). Put differently, although highly anxious followers tend to interact more often with narcissistic leaders, this effect is mitigated by the degree of leader popularity. Followers might interact with highly popular leaders regardless of whether such leaders are highly narcissistic. It is therefore reasonable that leader popularity might be an important factor to consider in the present study as well. Therefore, we created three types of sub-datasets. For each data set type, we dropped (a) the top 5% of all leader–follower interactions resulting in a data set in which only highly popular leaders were removed, (b) top 10% of all leader–follower interactions resulting in a data set in which highly popular and somewhat popular leaders are removed, or (c) top 15% of all leader–follower interactions resulting in a data set that only includes less popular leaders. Doing so allows us to interpret results based on the degree of leader popularity.
- *The ratio between followers’ friends count and follower count*: We also calculated the ratio between friends count and follower count (friend count/follower count^2^; Cresci et al., 2015). This ratio is the most important indicator of fake Twitter followers (Table 18; Cresci et al., 2015) and has been validated in previous research (Gruda, Karanatsiou, et al., 2021). A high friend-to-follower count ratio is indicative of bot-like behavior. Unfortunately, the exact cutoff to use for this ratio is unclear. We, therefore, examined our results based on two conditions, namely, (a) exclude followers with a ratio of either higher than 0.5 or (b) exclude followers with a ratio of higher than 1. We also tested the validity of using this ratio to detect bots by annotating a random sample of 1,000 Twitter followers on botometer (https://botometer.osome.iu.edu/api). Based on our random sample of 1,000 profiles, zero profiles were categorized as bot-like after our friends to follower count ratio cut-offs were implemented.

The results of our multiverse analyses will be interpreted in a fashion consistent with a random-effects model (Hamaker & Muthén, 2020). In a fixed-effects model, all multiverses would be assumed to be drawn by the same population. However, in a random-effects model, the various multiverses are assumed to be drawn from different populations. We choose to interpret the multiverse analyses as a random-effects model because we do believe that the aforementioned preprocessing decisions can affect the magnitude of the relationships. We can declare support for the hypothesized relationship if the coefficient in question has the same sign across all the multiverses and there is evidence that the majority of the multiverse coefficients are significant.

## Results

For purposes of our study, follower engagement was defined as the number of interactions between leaders and followers, that is, the number of follower replies per leader post. We used the Big Five personality traits and narcissism of both the leader and their followers to predict follower engagement. As followers are nested within leader groups and follower engagement was a count variable, we tested our hypotheses using multilevel mixed-effects Poisson regression. We confirm that for all multiverses, we have reported all measures, conditions, data exclusions, and we have explained how we determined the respective sample sizes. In addition, we follow the guidelines provided by Maas and Hox (2004), who stated that standard errors typically are estimated too small only in the case of fewer than 50 groups, with less than 30 observations per group. Given the parameters of all our data sets, we use normally distributed (standard) errors, specifying a random-effects model for all examined interactions.

**Figure 1.**
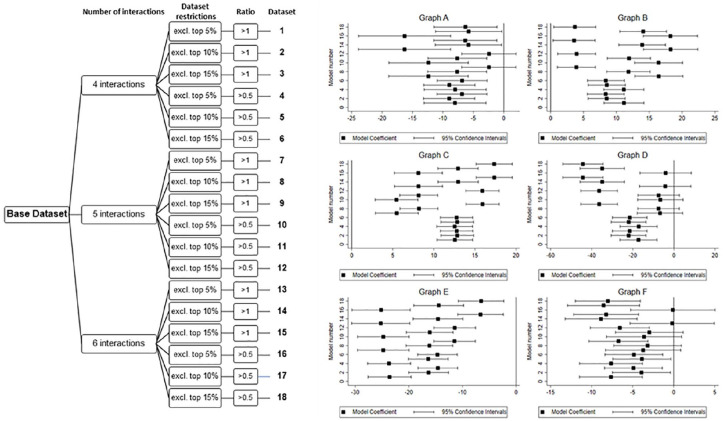
Decision tree and multiverse overview for all two-way interactions. *Note.* The decision tree on the left side shows how the multiverse of 18 data sets was created. The six panels on the right display the estimates and their 95% confidence intervals for each two-way interaction (i.e., leader narcissism and follower personality trait), resulting from multilevel modeling across the multiverse of 18 data sets; Graph A = Leader Narcissism X Follower Openness, Graph B = Leader Narcissism X Follower Agreeableness, Graph C = Leader Narcissism X Follower Neuroticism, Graph D = Leader Narcissism X Follower Narcissism, Graph E = Leader Narcissism X Follower Conscientiousness, Graph F = Leader Narcissism X Follower Extraversion; Ratio = Friends/Following^2^ for all followers.

**Figure 2.**
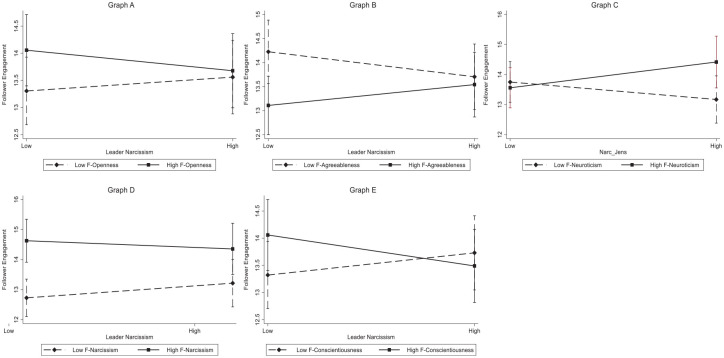
Graphical representation of results for selected significant two-way interactions. *Note.* Graphs represent independently two-way interaction regression models, control variables in each model incl. leader Big Five personality traits, follower Big Five personality traits, number of leader tweets, number of leader followers, number of leader user mentions; Graph A, B, and E = Model 16, Graph C and D = Model 18; F = Follower.

**Figure 3.**
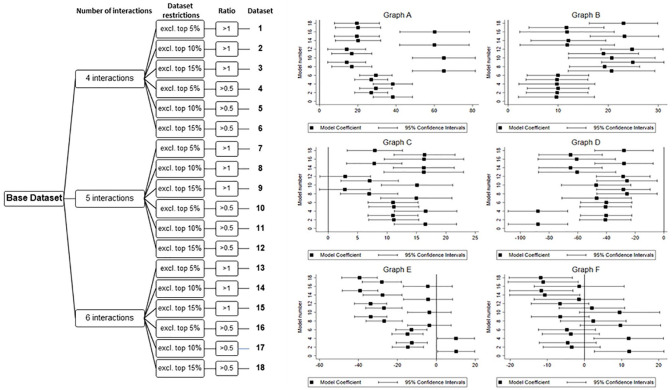
Decision tree and multiverse overview for selected three-way interactions. *Note.* The decision tree on the left side shows how the multiverse of 18 data sets was created. The six panels on the right display the estimates and their 95% confidence intervals for each three-way interaction (i.e., leader gender, leader narcissism, and follower personality trait), resulting from multilevel modeling across the multiverse of 18 data sets; Graph A = L-Gender X L-Narcissism X F-Openness, Graph B = L-Gender X L-Narcissism X F-Agreeableness, Graph C = L-Gender X L-Narcissism X F-Neuroticism, Graph D = L-Gender X L-Narcissism X F-Narcissism, Graph E = L-Gender X L-Narcissism X F-Conscientiousness, Graph F = L-Gender X L-Narcissism X F-Extraversion.

**Figure 4.**
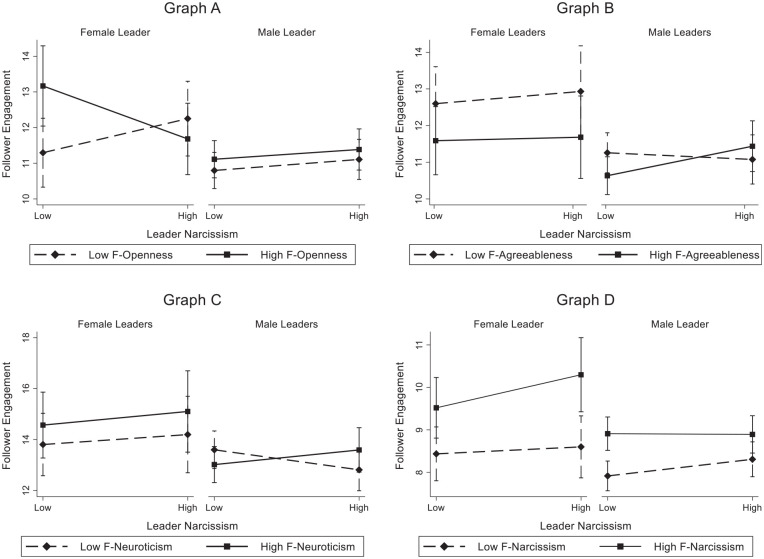
Graphical representation of results for selected significant three-way interactions. *Note.* Graphs represent independent three-way interactions and include additional controls incl. leader Big Five personality, follower Big Five personality, number of leader tweets, number of leader followers, number of leader user mentions; Graph A = Model 10, Graph B = Model 14, Graph C = Model 14, Graph D = Model 4; F = Follower.

An overview of the multiverse analysis regarding our main hypotheses is provided in Figure 1, while all two-way interactions across all multiverses are graphed in Figure 2 with model coefficients provided in Table 1. Multiverse analysis results by leader gender are provided in Figure 3, while respective three-way interactions are shown in Figure 4.

**Table 1.** Overview of Two-Way Interactions (Leader Narcissism and Follower Personality) on Follower Engagement.

Model	Interactions	Condition	Ratio	*#Leaders*	*#Followers*	L-NARC X F-OPEN		L-NARC X F-CONSCIENT		L-NARC X F-EXTRA		L-NARC X F-AGREE		L-NARC X F-NEUROT		L-NARC X F-NARC	
1	4	Excl. top 15%	>1	434	45,290	−8.02	[−13.11, −2.93]	−23.60	[−27.62, −19.59]	−7.66	[−11.49, −3.83]	11.15	[8.11, 14.19]	12.55	[10.37, 14.73]	−17.24	[−26.28, −8.2]
2	4	Excl. top 10%	>1	435	47,465	−8.98	[−13.19, −4.77]	−16.32	[−20.04, −12.6]	−3.93	[−7.47, −0.39]	8.52	[5.7, 11.33]	12.85	[10.87, 14.84]	−22.05	[−30.5, −13.59]
3	4	Excl. top 5%	>1	436	49,674	−6.87	[−10.98, −2.76]	−14.61	[−18.31, −10.91]	−4.91	[−8.42, −1.39]	8.38	[5.58, 11.18]	12.77	[10.8, 14.75]	−21.55	[−29.98, −13.13]
4	4	Excl. top 15%	>0.5	434	45,263	−8.00	[−13.09, −2.91]	−23.71	[−27.73, −19.69]	−7.64	[−11.47, −3.81]	11.14	[8.09, 14.18]	12.53	[10.35, 14.71]	−17.17	[−26.21, −8.13]
5	4	Excl. top 10%	>0.5	435	47,435	−8.95	[−13.17, −4.74]	−16.40	[−20.12, −12.68]	−3.88	[−7.42, −0.34]	8.54	[5.72, 11.36]	12.84	[10.85, 14.83]	−22.03	[−30.48, −13.57]
6	4	Excl. top 5%	>0.5	436	49,644	−6.85	[−10.96, −2.74]	−14.68	[−18.38, −10.98]	−4.85	[−8.37, −1.33]	8.40	[5.6, 11.21]	12.76	[10.78, 14.74]	−21.53	[−29.95, −13.1]
7	5	Excl. top 15%	>1	314	26,988	−12.39	[−18.92, −5.86]	−24.79	[−29.59, −19.98]	−3.73^†^	[−8.31, 0.85]	16.46	[12.8, 20.11]	5.48	[2.88, 8.08]	−6.79^†^	[−17.86, 4.28]
8	5	Excl. top 10%	>1	315	28,477	−7.66	[−12.5, −2.83]	−16.16	[−20.52, −11.81]	−3.19^†^	[−7.31, 0.93]	11.83	[8.53, 15.12]	8.18	[5.87, 10.49]	−7.46^†^	[−17.57, 2.64]
9	5	Excl. top 5%	>1	317	32,256	−2.46^†^	[−6.93, 2]^†^	−11.49	[−15.36, −7.62]	−6.75	[−10.35, −3.14]	3.92	[1.03, 6.81]	15.92	[13.91, 17.94]	−36.36	[−45.27, −27.46]
10	5	Excl. top 15%	>0.5	314	26,980	−12.37	[−18.9, −5.84]	−24.78	[−29.59, −19.97]	−3.62^†^	[−8.2, 0.96]	16.42	[12.77, 20.07]	5.45	[2.85, 8.05]	−6.62^†^	[−17.69, 4.45]
11	5	Excl. top 10%	>0.5	315	28,466	−7.63	[−12.47, −2.8]	−16.12	[−20.48, −11.76]	−2.97^†^	[−7.09, 1.15]	11.90	[8.61, 15.2]	8.14	[5.84, 10.45]	−7.41^†^	[−17.52, 2.7]
12	5	Excl. top 5%	>0.5	317	32,243	−2.44^†^	[−6.91, 2.02]	−11.46	[−15.33, −7.59]	−6.57	[−10.17, −2.97]	3.96	[1.07, 6.85]	15.90	[13.88, 17.92]	−36.39	[−45.3, −27.48]
13	6	Excl. top 15%	>1	238	18,865	−16.32	[−23.9, −8.74]	−25.25	[−30.67, −19.83]	−0.19^†^	[−5.34, 4.95]	18.26	[14.13, 22.38]	8.13	[5.21, 11.05]	−4.24^†^	[−16.72, 8.24]
14	6	Excl. top 10%	>1	240	21,221	−5.80	[−11.26, −0.35]	−14.57	[−19.23, −9.92]	−8.85	[−13.24, −4.47]	13.92	[10.38, 17.46]	12.95	[10.51, 15.39]	−34.90	[−45.67, −24.14]
15	6	Excl. top 5%	>1	241	23,065	−6.33	[−11.51, −1.15]	−6.64	[−10.89, −2.39]	−8.24	[−12.2, −4.28]	3.57	[0.37, 6.76]	17.35	[15.15, 19.55]	−44.32	[−54.1, −34.54]
16	6	Excl. top 15%	>0.5	238	18,859	−16.31	[−23.89, −8.74]	−25.21	[−30.64, −19.78]	−0.11^†^	[−5.25, 5.03]	18.21	[14.09, 22.34]	8.11	[5.19, 11.03]	−4.04^†^	[−16.52, 8.44]
17	6	Excl. top 10%	>0.5	240	21,211	−5.76	[−11.22, −0.31]	−14.45	[−19.1, −9.79]	−8.55	[−12.94, −4.16]	14.07	[10.53, 17.62]	12.93	[10.49, 15.38]	−34.91	[−45.68, −24.14]
18	6	Excl. top 5%	>0.5	241	23,055	−6.30	[−11.48, −1.12]	−6.53	[−10.78, −2.28]	−8.03	[−11.99, −4.07]	3.67	[0.48, 6.86]	17.34	[15.14, 19.54]	−44.31	[−54.09, −34.53]

*Note.* Table displays two-way interaction (unstandardized) coefficients and corresponding 95% CIs in brackets across all examined 18 created multiverses; # = Number of; L-NARC = Leader Narcissism, F-OPEN = Follower Openness, F-CONSCIENT = Follower Conscientiousness, F-EXTRA = Follower Extraversion, F-AGREE = Follower Agreeableness, F-NEUROT = Follower Neuroticism, F-NARC = Follower Narcissism); Ratio = Friends/Following^2^ for all followers; CI = confidence interval.

All interaction coefficients are significant except coefficients marked with ^†^*p* > .05.

### Leader Narcissism and Follower Personality Traits

H1 stated that the leader narcissism–follower engagement relationship will be moderated by follower openness. Specifically, the magnitude of this relationship will be negatively related to followers’ openness to experience. As can be seen in Graph A of Figure 1 and consistent with H1, the interaction was negative for all of the 18 multiverses. Except for two models (i.e., Models 9 and 12), the confidence intervals (CIs) for the multiverses did not include zero. Given that the coefficients for all of the multiverses were in the predicted direction and that these coefficients were significantly different from zero for 16 of the 18 (88.9%) multiverses, H1 was supported.

We plotted this interaction using the results from Multiverse 16 and this interaction is shown in Graph A of Figure 2. Followers who were open to experience were less engaged as leader narcissism increased. Followers who were less open to experience were more engaged as leader narcissism increased. Interestingly, both multiverses whose CIs included zero included very popular leaders (i.e., the data in both of these multiverses only removed the top 5% of leaders). Overall, H1 was supported but the strength of this interaction was stronger when the most popular leaders were excluded from the analyses.

H2 predicted that the leader narcissism–follower engagement relationship will be moderated by follower agreeableness. Specifically, the magnitude of this relationship will be positively related to follower agreeableness. Consistent with this hypothesis, Graph B of Figure 1 shows that the interaction coefficients were positive and significant for all multiverses. We plotted this interaction for Multiverse 16 and this plot is shown in Graph B of Figure 2. Agreeable followers were more engaged as leader narcissism increased, whereas less agreeable followers were less engaged as leader narcissism increased. In summary, H2 was supported across all 18 multiverses.

H3 predicted that follower neuroticism will moderate the leader narcissism–follower engagement relationship. Specifically, the magnitude of this relationship will be positively related to follower neuroticism. As seen in Graph C of Figure 1, the interaction coefficients were positive and significant for all multiverses. We plotted this interaction for Multiverse 16 (shown in Graph C of Figure 2). There was a positive relationship between leader narcissism and follower engagement for highly neurotic followers. Specifically, highly neurotic followers were more engaged as leader narcissism increased. Followers who were less neurotic became less engaged as leader narcissism increased. Thus, H3 was supported.

H4 predicted that the relationship between leader narcissism and follower engagement would be moderated by follower narcissism. Specifically, the magnitude of this relationship will be negatively related to follower narcissism. As shown in Graph D of Figure 1, the interaction coefficients were negative for all multiverses but significant in 12 of the 18 multiverses (66.7%). Although there was weaker support for this hypothesis compared with the others, we decided to plot it anyway using Multiverse 16. As seen in Graph D of Figure 2, followers lower in narcissism became more engaged as leader narcissism increased. There did not appear to be a relationship between leader narcissism and follower engagement for followers higher in narcissism. Thus, there is weak support for H4.

Although we did not state specific hypotheses for the moderating effect of either follower conscientiousness or follower extraversion on the leader narcissism–follower engagement relationship, we nonetheless visualize these results across the 18 multiverses to provide a holistic overview of our results. Concerning the results for follower conscientiousness (Graph E of Figure 1), we found that the coefficients were negative and significant for all 18 multiverses. Graph E of Figure 2 shows the interaction for Multiverse 16. The positive relationship between leader narcissism and follower engagement decreased and became negative as follower conscientiousness increased.

Finally, Graph F of Figure 1 shows the results regarding the moderating effect of follower extraversion. Negative interaction coefficients were found for all of the multiverses, but this interaction was nonsignificant in 12 of the 18 multiverses (66.7%). Based on the number of multiverses with nonsignificant interactions, we conclude that we do not find support for a moderating effect of follower extraversion. Given this conclusion, we refrained from graphing this interaction.

### The Moderating Role of Gender

Finally, H5 predicted that there will be a three-way interaction on follower engagement as a function of leader gender, leader narcissism, and follower personality characteristics, given the documented stereotype about females and leadership ability. Specifically, we predicted that the magnitude of this relationship will be larger for males than for females. An overview of the multiverse results of the various three-way interactions is provided in Figure 3 with the coefficients for all the multiverses provided in Table 2. Figure 4 shows the nature of the three-way interactions.

**Table 2.** Overview of Three-Way Interactions (Leader Gender, Leader Narcissism, and Follower Personality) on Follower Engagement.

Model	#Interactions	Condition	Ratio	*#Leaders*	*#Followers*	Male Leaders %	Female Leaders %	L-Gender X NARC X F-OPEN		L-Gender—NARC X F-CONS		L-Gender—NARC X F-EXTRA		L-Gender—NARC X F-AGREE		L-Gender—NARC X F-NEUROT		L-Gender—NARC X F-NARC	
1	4	Excl. top 15%	>1	434	45,290	75.12	24.88	38.3	[28.05,48.56]	10.14	[0.61, 19.67]	12.03	[2.68, 21.38]	9.65	[2.07, 17.24]	16.51	[11.2, 21.81]	−87.77	[−108.53, −67.01]
2	4	Excl. top 10%	>1	435	47,465	74.94	25.06	27.04	[18.41,35.67]	−14.66	[−22.68, −6.64]	−3.44^†^	[−11.11, 4.24]	9.79	[3.63,15.96]	11.14	[6.88, 15.4]	−40.8	[−58.7, −22.91]
3	4	Excl. top 5%	>1	436	49,674	75	25	29.46	[20.95,37.97]	−12.63	[−20.63, −4.63]	−4.51^†^	[−12.17, 3.15]	9.98	[3.82, 16.13]	11	[6.75, 15.25]	−40.3	[−58.18, −22.42]
4	4	Excl. top 15%	>0.5	434	45,263	75.12	24.88	38.29	[28.03,48.54]	10.04	[0.5, 19.58]	11.89	[2.52, 21.26]	9.74	[2.16, 17.33]	16.56	[11.25, 21.86]	−87.82	[−108.59, −67.05]
5	4	Excl. top 10%	>0.5	435	47,435	74.94	25.06	27.04	[18.4,35.67]	−14.84	[−22.86, −6.81]	−3.66^†^	[−11.35, 4.02]	9.77	[3.59, 15.94]	11.15	[6.89, 15.41]	−40.67	[−58.57, −22.77]
6	4	Excl. top 5%	>0.5	436	49,644	75	25	29.45	[20.94,37.96]	−12.81	[−20.82, −4.8]	−4.73^†^	[−12.4, 2.94]	9.95	[3.79, 16.11]	11.02	[6.76, 15.27]	−40.17	[−58.05, −22.28]
7	5	Excl. top 15%	>1	314	26,988	75.16	24.84	64.99	[48.54,81.44]	−3.61^†^	[−14.78, 7.56]	9.67^†^	[−1.13, 20.47]	20.67	[12.01, 29.32]	14.97	[8.91, 21.03]	−46.91	[−71.07, −22.74]
8	5	Excl. top 10%	>1	315	28,477	74.92	25.08	16.87	[6.41,27.34]	−26.81	[−36.14, −17.48]	2.37^†^	[−6.45, 11.18]	19.28	[12.28, 26.28]	6.96	[2.08, 11.84]	−25.78	[−46.82, −4.74]
9	5	Excl. top 5%	>1	317	32,256	74.76	25.24	14.21	[4.25,24.17]	−33.71	[−42, −25.41]	−6.52^†^	[−14.28, 1.25]	24.91	[18.69, 31.13]	2.83^†^	[−1.48, 7.14]	−28.28	[−46.97, −9.6]
10	5	Excl. top 15%	>0.5	314	26,980	75.16	24.84	64.99	[48.54,81.45]	−3.62^†^	[−14.81, 7.56]	9.45^†^	[−1.35, 20.25]	20.7	[12.05, 29.36]	15.09	[9.03, 21.15]	−47.24	[−71.41, −23.06]
11	5	Excl. top 10%	>0.5	315	28,466	74.92	25.08	16.85	[6.39,27.31]	−26.93	[−36.27, −17.59]	2.0^†^	[−6.82, 10.82]	19.06	[12.05, 26.06]	7.04	[2.16, 11.92]	−25.76	[−46.81, −4.71]
12	5	Excl. top 5%	>0.5	317	32,243	74.76	25.24	14.21	[4.24,24.17]	−33.78	[−42.09, −25.48]	−6.58^†^	[−14.34, 1.19]	24.76	[18.54, 30.98]	2.89^†^	[−1.42, 7.19]	−28.43	[−47.12, −9.74]
13	6	Excl. top 15%	>1	238	18,865	74.37	25.63	60.07	[41.89,78.25]	−4.26^†^	[−16.82, 8.3]	−1.48^†^	[−13.57, 10.61]	11.81	[2.38, 21.25]	16.24	[9.47, 23]	−60.51	[−87.48, −33.55]
14	6	Excl. top 10%	>1	240	21,221	74.17	25.83	20.13	[8.3,31.97]	−27.67	[−37.67, −17.66]	−10.67	[−20.1, −1.24]	12.04	[4.56, 19.52]	16.24	[11.05,21.42]	−65.12	[−87.23, −43.01]
15	6	Excl. top 5%	>1	241	23,065	73.86	26.14	19.52	[7.77,31.27]	−39.19	[−48.29, −30.08]	−11.61	[−20.12, −3.1]	23.25	[16.42, 30.08]	7.82	[3.12,12.51]	−27.84	[−48.19, −7.5]
16	6	Excl. top 15%	>0.5	238	18,859	74.37	25.63	60.18	[42,78.36]	−4.41^†^	[−16.99, 8.18]	−1.42^†^	[−13.51, 10.67]	11.77	[2.34, 21.2]	16.28	[9.52,23.05]	−60.82	[−87.81, −33.83]
17	6	Excl. top 10%	>0.5	240	21,211	74.17	25.83	20.12	[8.29,31.96]	−28.01	[−38.03, −17.99]	−11.11	[−20.54, −1.67]	11.66	[4.18, 19.14]	16.36	[11.18,21.55]	−65.08	[−87.19, −42.96]
18	6	Excl. top 5%	>0.5	241	23,055	73.86	26.14	19.52	[7.77,31.28]	−39.45	[−48.57, −30.33]	−11.77	[−20.28, −3.26]	23.05	[16.22, 29.89]	7.92	[3.22,12.62]	−27.86	[−48.22, −7.51]

*Note.* Table displays three-way interaction (unstandardized) coefficients and corresponding 95% CIs in brackets across all examined 18 created multiverses; # = Number of; L-Gender = Leader Gender, L-NARC = Leader Narcissism, F-OPEN = Follower Openness, F-CONSCIENT = Follower Conscientiousness, F-EXTRA = Follower Extraversion, F-AGREE = Follower Agreeableness, F-NEUROT = Follower Neuroticism, F-NARC = Follower Narcissism); Ratio = Friends/Following^2^ for all followers.

All interaction coefficients are significant (*p* < .05) except coefficients marked with ^†^*p* > .05.

Figure 3 shows that, across all the multiverses, the interactions between leader gender, leader narcissism, and follower (a) openness, (b) agreeableness, and (c) narcissism were robust across all multiverses. That is, the three-way interactions were significantly different from zero and the sign of the interaction’s coefficients was all in the same direction for all of the 18 multiverses. The three-way interaction between leader gender, leader narcissism, and openness is shown in Graph A of Figure 3. As shown in this graph, the interaction’s coefficients were positive for all multiverses. We plotted the three-way interaction for Multiverse 10 and this is shown in Graph A of Figure 4. In contrast to H5, the three-way interaction was evident for female as opposed to male leaders. For female leaders, high openness followers were less likely to engage as leader narcissism increased. However, for followers low in openness, engagement increased as the narcissism of female leaders increased. There was no apparent interaction for male leaders.

Consistent with H5, the three-way interaction between leader narcissism, follower agreeableness, and leader gender was significant across all multiverses and was all positive (see Graph B in Figure 3). We plotted this three-way interaction for Multiverse 12. As shown in Graph B in Figure 4, highly agreeable followers were more likely to engage as narcissism increased for male leaders. Followers lower in agreeableness were less likely to engage as narcissism increased for male leaders. There was no apparent interaction for female leaders.

Similarly, the results for the three-way interaction leader gender, leader narcissism, and follower neuroticism were consistent with H5. As shown in Graph C of Figure 3, the three-way interactions were positive for all multiverses and were significant for 16 of the 18 (88.9%) multiverses. Graph C in Figure 4 shows this three-way interaction for Multiverse 14. Highly neurotic followers were more likely to engage as narcissism increased for male leaders. Less neurotic followers were less likely to engage as narcissism increased for male leaders. There was no apparent interaction for female leaders.

Finally, the three-way interaction between leader gender, leader narcissism, and follower narcissism did not support H5. As shown in Graph D of Figure 3, the interactions were significant and negative for all the multiverses. We plotted this interaction using Multiverse 4 and this plot is shown in Graph D of Figure 4. For female leaders, the more narcissistic followers were engaged as narcissism in the leader increased. No apparent relationship between leader narcissism and follower engagement was found for female leaders and less narcissistic followers. Interestingly, this pattern reversed for male leaders. For male leaders, the less narcissistic followers were more engaged as narcissism in the leader increased. No such pattern existed for narcissistic followers with male leaders.

Finally, Graph E of Figure 3 shows the three-way interaction with follower conscientiousness and Graph F of Figure 3 shows the three-way interaction with follower extraversion. As can be seen in these graphs, the three-way interaction coefficients were both positive and negative across the 18 multiverses. In addition, the interaction was significant in only eight multiverses in the case of follower extraversion. Given the lack of consistency for the direction of these three-way interactions, we concluded that leader gender did not interact with these follower personality characteristics. Therefore, we did not plot these three-way interactions.

Overall, on one hand, it seems that while leader gender was important to interpret our results, H5 stated that the interaction pattern would be stronger for male leaders as opposed to female leaders. This predicted interaction pattern was found for follower agreeableness and neuroticism. For these follower attributions, larger changes in follower engagement were found for male as opposed to female leaders. On the other hand, in the case of follower openness and follower narcissism, larger changes in follower engagement were found for female as opposed to male leaders. We discuss these results in the following section.

## Discussion

Although research interest in the dark triad traits, and in particular, leader narcissism, has been on the rise over the past few years (Grijalva, Harms, et al., 2015), prior literature has predominantly discussed leader narcissism from a leader-centric perspective. Although it is important in understanding how leader characteristics shape leader–follower relationships, the follower’s perspective of leader narcissism should not be ignored. Understanding which followers are more likely to be drawn to narcissistic leaders is vital in gaining a holistic overview of this topic (Gruda, Karanatsiou, et al., 2021) and in improving leader–follower relationships, team formation, as well as recruitment and selection of both leaders and followers. Examining the interaction between follower personality traits and leader narcissism using a large sample of observations and interactions on the Twitter platform, on the leader as well as the follower level, is a step in this direction.

One major contribution of this study is that we performed multiverse analyses^3^ to understand the robustness of our results across the various preprocessing decisions that were made to the base data set. Our multiverse analyses examined the extent to which our conclusions were limited by the number of leader–follower interactions as well as various degrees of leader popularity. For example, it is possible that in the short term, narcissistic followers interact more with highly narcissistic leaders compared with less narcissistic leaders. Therefore, we would expect the frequency of interactions to drop after these initial exchanges, as followers realize the degree of narcissism exhibited by the leader. The presented multiverse analysis provided greater clarity in this regard, as we showcased results for multiple leader–follower interaction conditions, namely, four, five, and six interactions. We found that regardless of the minimum number of leader–follower interactions, followers who score high on agreeableness and neuroticism were more likely to engage with narcissistic leaders, whereas followers who score low on openness to experience interacted less often with narcissistic leaders, compared with non-narcissistic leaders. Hence, it seems that, regardless of the number of interactions, followers who are dispositioned to be agreeable or neurotic are likely prone to be more susceptible to narcissistic leaders, likely due to these leaders’ charismatic charm and confident behavior (Rosenthal & Pittinsky, 2006). These findings provide support for our hypotheses (H1–H3).

In addition, the presented multiverse analysis also allows us to account for varying degrees of leader popularity. Previous studies (e.g., Gruda, Karanatsiou, et al., 2021) found that the relationship between leader narcissism and follower traits, namely, trait anxiety, differs based on observed leader popularity. By restricting our data set in one of three ways (i.e., by excluding the top 5%, 10%, or 15% of all follower observations), we effectively created multiple data sets that include very popular leaders (i.e., leaders with a lot of followers), popular leaders, and less popular leaders. These alternate paths allow us to draw conclusions that would not have been possible beforehand. For example, we found more limited support for the relationship between leader narcissism and follower narcissism (H4). In the case of a low number of leader–follower interactions (Figure 2, Models 1–6), it seems that non-narcissistic followers seem to be more likely to engage with narcissistic leaders. However, as the number of interactions increases (Figure 2, Models 7–8 and 10–11), this effect weakens. Likewise, it seems that leader popularity also plays a role, as the strongest effect was observed in the most inclusive models (i.e., models that only exclude the top 5% of all observations). It might be the case that as leaders gain more popularity, non-narcissistic followers are more likely to follow and engage with them, regardless of the observed degree of leader narcissism.

Finally, although not initially hypothesized, we found a significant and negative interaction between leader narcissism and follower conscientiousness (Figure 2). Hence, it seems that less conscientious followers interact more often with narcissistic leaders, compared with highly conscientious followers. And although the observed model coefficients all differed from zero and the effect was unidirectional (and negative), this particular relationship seems to be largely influenced by both the number of leader–follower interactions and leader popularity. Put differently, as the number of leader–follower interactions increases, the less likely highly conscientious followers are to engage with narcissistic leaders. Likewise, the largest effects were observed in models that were based on less popular leaders (i.e., models excluding the top 15% of all observations). Hence, it seems that as leaders gain more popularity, conscious followers are more likely to engage with them regardless of the observed degree of leader narcissism.

### The Moderating Role of Leader Gender

We also found a significant interaction between leader gender, leader narcissism, and follower personality traits, namely, openness, agreeableness and neuroticism, and narcissism. First, concerning followers who scored high on openness to experience, we observed a significant effect in the case of female leaders. It seems that followers who score low on openness to experience tend to interact more often with narcissistic female leaders compared with non-narcissistic female leaders. The same effect does not seem to be the case in male leaders.

Second, highly agreeable or neurotic followers seemed to interact more often with narcissistic male leaders compared with non-narcissistic male leaders. Hence, for both of these followers’ personality traits, significant effects were mostly only in the case of male leaders. This provides evidence that highly agreeable or neurotic followers are more likely to endorse and interact with male narcissistic leaders, as hypothesized (H4).

Finally, narcissistic followers seemed to interact more often with female narcissistic leaders, whereas non-narcissistic followers were more likely to interact with male narcissistic leaders. These results are quite interesting, as they highlight the importance of possible gender moderation effects in leader–follower relationships. However, it is important to keep in mind that we did not account for follower gender differences given that Twitter does not allow the direct collection of user demographic information. It might be that follower gender and leader–follower gender congruence influence the perception of (male and female) narcissistic leaders differently. This would be an important point to examine in future studies.

For example, Thoroughgood et al. (2011) found that followers’ perceptions of their leaders depend heavily on the corporate climate and financial performance, as well as their leaders’ gender. Female leaders who broke the rules were perceived more negatively than male leaders, but only in companies intolerant of aversive leadership and companies that were exhibiting negative organizational performance. Hence, these two factors seem to be not simply distinct moderating effects, but rather a three-way interaction between gender and industry might moderate followers’ perceptions of their leader. It might be interesting to examine this three-way interaction further (Padilla et al., 2007; Spain et al., 2014) in subsequent studies.

## Implications

There is growing interest in followership. Unfortunately, most of this literature is primarily focuses on building taxonomies of different styles of followers (Chaleff, 2009; Kellerman, 2008). The different followership models may provide some suggestive hints about the underlying mechanisms that account for different follower behavior. For example, it has been suggested that the style of followership may be determined by followers’ engagement level (Kellerman, 2008), followers’ courage (Chaleff, 2009), or the situational context followers are placed into (Bjugstad et al., 2006). The focus of these followership models always is on classifying different follower behavior and not on the underlying mechanisms that might explain these behaviors.

In contrast to these followership models, the present study focused on one mechanism (i.e., personality and narcissism levels of leader and follower) and empirically tested whether the interaction of these characteristics can account for follower behavior (i.e., engagement). We found support for the interaction between leader characteristics (i.e., narcissism and gender) and follower characteristics (i.e., Big Five personality and narcissism) in understanding an important behavior in the leader–follower relationship (i.e., follower engagement). Of course, this is only one mechanism and it is unlikely that the range of the identified follower behavioral categories can be adequately explained by this single mechanism. Other mechanisms need to be explored, such as leaders’ and followers’ needs, communication styles, and attachment orientations (Gruda & Kafetsios, 2020) to more completely understand why different followership styles emerge.

Another implication is that the present study demonstrated the utility in expanding the context used to study leadership. We identified the overlap in influence strategies used by leaders in brick-and-mortar organizations and virtual contexts. While researchers may have originally questioned whether this study would generalize to more traditional organizations, an equally valid question is whether the prior leadership and followership literature are generalizable to the virtual workplace and interactions. Many organizations survived the COVID-19 pandemic by turning to social media and virtual environments to continue their businesses. The shift in the business model caused by the pandemic not only affected organizations but also affected workers. As a result of the pandemic and the reliance on virtual workplaces, a migration of the population has occurred in the United States due to workers discovering that they could successfully maintain their job even if they move far away from their work. It is still too early to know the long-term implications of this transformation of the workforce, but as of this writing, there is still a shortage of workers due to the resistance of people to go back to less than desirable jobs.

Finally, the political upheaval that occurred in the United States in 2020 and 2021 was a result of certain individuals using social media to lead masses of followers to either take actions (e.g., January 6th attack on the U.S. Congress; Black-Lives-Matter movement) or not take actions (e.g., refusing COVID-19 vaccine). The power of leadership in a virtual environment has been documented by the actual political and social events that occurred in the recent past. This study has documented that virtual leadership can be meaningfully studied.

## Conclusion

We provide a follower-centric view of leader narcissism based on online interactions between leaders and followers on the Twitter platform using an ML and multiverse analytical approach. We find that followers who score high on certain personality traits, including agreeableness and neuroticism, are most likely to engage with narcissistic leaders. We also examine and present results of the moderation effects of leader gender.

## Supplemental Material

sj-docx-1-psp-10.1177_01461672221094976 – Supplemental material for Don’t Go Chasing Narcissists Click here for additional data file. Supplemental material, sj-docx-1-psp-10.1177_01461672221094976 for Don’t Go Chasing Narcissists by Dritjon Gruda, Dimitra Karanatsiou, Paul Hanges, Jennifer Golbeck and Athena Vakali in Personality and Social Psychology Bulletin

## Notes

1. A minimal version of our data set is available here: https://osf.io/xzs8w/

2. It should be noted that narcissism is composed of two types, namely, grandiose and vulnerable narcissism (Clifton, 2011). In this article, we exclusively focus on grandiose narcissism. Grandiose narcissism is associated with being overconfident, having a positively distorted view of self and feelings of entitlement. Such individuals, at least in the short run, can be perceived as charming and impressive. However, in the long run, such perceptions can fade.

3. We would like to thank the anonymous reviewer for this suggestion.
