# Supplementary material for: Don’t Go Chasing Narcissists: A Relational-Based and Multiverse Perspective on Leader Narcissism and Follower Engagement Using a Machine Learning Approach
Source: Pers Soc Psychol Bull. 2022 May 27;49(7):1130–47. doi: 10.1177/01461672221094976 (PMC10302363; doi:10.1177/01461672221094976)
Supplement: sj-docx-1-psp-10.1177_01461672221094976 – Supplemental material for Don’t Go Chasing Narcissists [file sj-docx-1-psp-10.1177_01461672221094976.docx]

**Supplementary Material**

**Personality and narcissism annotation**

To estimate personality and narcissism scores for individuals in our dataset, we applied a pre-trained predictive model and developed a novel ML model, respectively. Applying models that are trained in a similar context, for example, NLP tasks, Twitter users profiles, etc., present the great benefit of annotating with high levels of reported accuracy large amounts of data, while the acquisition of human-labeled data (self-reported questionnaires in the case of narcissism and personality traits) can be an expensive task (Han et al., 2021). State-of-the-art machine learning methods allow researchers to transfer learning in different environments if the main task-goal remains the same. Since the environment (Twitter) and the available information to be extracted is similar, applying the pre-trained model to label our dataset with personality scores consists of a valid and suitable practice. Descriptive statistics of the ground truth dataset for the Big Five personality traits used to build upon the pre-trained model can be found in Table S1.

-------------------------------------------

Insert Table S1 about here

-------------------------------------------

After collecting tweet history and profile attributes, we followed preprocessing steps to maintain a clean and noise-free dataset and subsequently extracted insightful features that act as a proxy of personality traits. After extracting relevant features, we applied a pre-trained model (Gruda et al., 2020) to annotate leaders and followers with Big Five personality scores, which provided a fairly high prediction accuracy with a Mean Squared Error of .066. In addition, we developed an algorithm able to predict the narcissism scores of Twitter users with MSE .081. The detailed process is described as follows:

1. **Collecting the ground-truth dataset (Participants and procedures).** Ground truth or empirical evidence is an important component in ML as it is used for model training. We acquired the ground truth data by distributing an online survey. An article on narcissism was published on Psychology Today’s webpage (Golbeck, 2014; psychologytoday.com). At the end of the article, a link connected interested participants to a different webpage to complete a short survey. Participants read the informed consent statement and answered a single-item measure of narcissism (Konrath et al., 2014). Specifically, participants were asked to indicate their agreement on the following statement “To what extent do you agree with this statement: ‘I am a narcissist? (Note: The word ‘narcissist’ means egotistical, self-focused, and vain)” by using a 7-point (1 = “Not Very True of Me” to 7 = “Very True of Me”) Likert scale. The survey also asked for participants’ Twitter usernames. A total of 1,067 participants answered the narcissism item (range: 1-7; M = 3.00, SD = 1.606), provided their public (and valid) Twitter account name, and provided consent for their data to be used for research purposes.
2. **Training the narcissism model.** After collecting users' tweets, we followed pre-processing and features extraction steps. In particular, we perform text cleaning, filtering out noisy information (i.e URLs, etc), tokenization, and part of speech tagging. After this process, we extracted text-related features to capture language expression in the form of unexpected patterns of vocabulary usage and phrases adoption with Tf-Idf and Ngrams vectors, employing an open vocabulary approach. Term frequency-inverse document frequency (Tf–Idf) is a term scoring method used in information retrieval and reflects the importance of a word to a document in a collection or corpus. Specifically, the more often a word appears in a document, the more it is considered to be significant (Aizawa, 2003). N-grams representations consist of a statistical measure of the frequency of words and sequences of words (phrases) usage. In the field of computational linguistics, an Ngram is any sequence of (contiguous) words in a text. For example, the 3-gram sequences that arise from the sentence “Twitter is a microblogging platform” are (Twitter, is, a), (is, a, microblogging), (a, microblogging platform). To account for more targeted features linked with narcissistic tendencies, we also extracted i-talk features, a set of words that refers to one’s self for example I, me, myself, mine, that are more likely to be used extensively by narcissistic personalities (Bollaert et al., 2019).

Finally, to account for behavioral patterns of users we employed Twitter attributes and platform metrics such as the number of followers, number of user mentions, frequency of status updates, and other relevant indicators^[[1]](#footnote-1)^ as outlined in Gruda et al. (2020). An overview of all features used in model training to build the narcissism regressor is provided in Table S2.

-------------------------------------------

Insert Table S2 about here

-------------------------------------------

As can be seen from the provided information in the paper and the response, the model does not rely on factors such as SES, age, and gender. While these factors could be interesting to include for future research purposes, the provided ground truth dataset only asked participants to indicate their degree of narcissism using the respective narcissism measure and indicate their public Twitter profile information. Hence, this information was not included in our model. Instead, we relied on linguistic and behavioral user residue based on social media information.

To adopt the best-performing model, we split the original dataset into train and test sets, while keeping 80% of the data for training and 20% for testing purposes. We followed a 10 fold cross-validation scheme in our training for hyperparameters tuning and experimentation, and the final selected model was tested on the test set. After experimenting with different regression algorithms namely linear, support vector machine, and random forest, we concluded on employing Random Forest as the best performing compared to other algorithms. A model comparison is provided in Table S3.

-------------------------------------------

Insert Table S3 about here

-------------------------------------------

Random Forest is a meta-estimator using the prediction of decision trees as the mean output score. This approach has the benefits of enhancing predictive accuracy and controlling potential over-fitting (Breiman, 2001). We conducted a grid search for the tuning of the hyperparameters of the RF regressors, which led to 100 trees and followed an early pruning strategy to reduce overfitting (Liaw & Wiener, 2002). Model performance was evaluated in the test set using the Mean Squared Error (MSE).

Finally, we acknowledge that the number of followers is one of several of the components of the narcissism model, which is related to our dependent variable (i.e., follower engagement). However, we do control for leaders’ number of followers as well as leaders’ numbers of posts in all models presented in the multiverse analysis. Doing so allows us to, at least partially, separate out and account for the effects of to such variables.

**Table S1**

Ground truth dataset descriptive statistics for Big Five personality trait inference algorithm

|  | M | SD | Min | Max |
| --- | --- | --- | --- | --- |
| Openness | 4.10 | 0.77 | 1.00 | 5.00 |
| Conscientiousness | 3.69 | 0.89 | 1.00 | 5.00 |
| Extraversion | 2.65 | 1.09 | 1.00 | 5.00 |
| Agreeableness | 3.89 | 0.84 | 1.00 | 5.00 |
| Neuroticism | 2.45 | 1.06 | 1.00 | 5.00 |

**Table 2**

Features used in model training in order to build the narcissism regressor

| **Features Category** | **Indicators** | **Description** |
| --- | --- | --- |
| **Language vectors (open vocabulary approach)** | *Tfidf vectors* | *a numerical statistic that is intended to reflect how important a word is to a document in a collection or corpus* |
|  | *Ngram vectors* | *how often words and phrases are used in a collection or corpus* |
| **Narcissism related indicators** | *italk* | *a set of words that refers to one’s self* |
| **Behavioral Attributes** | *Followers count* | number of followers a user has |
|  | *Favorite count* | number of items a user marked as favorite |
|  | *Statuses count* | number of statuses a user has posted |
|  | *List count* | number of lists a user appears on |
|  | *Days at Twitter* | number of days an individual uses the service, reflecting their experience in the platform |
|  | *Frequency of status updates* | calculated by the division of the number of statuses with the days at twitter, reflecting how active a user is |
|  | *No of links* | number of external links a user has shared in the platform |
|  | *No of hashtags* | number of hashtags a user has used to participate in public conversations |
|  | *No of user mentions* | number of times a user has mentioned another Twitter user |
|  | *No of retweets* | number of times a user has retweeted another users’ content |
|  | *Screen name length* | number of characters a user uses as their screen name |
|  | *Description length* | number of characters a user uses to describe himself |
|  | *Average tweet-length* | average length of each user tweet |
|  | *Average words* | average number of words a user uses on their tweets |
|  | *Average upper letter words* | average number of upper letter words in tweets reflect intense emotions and yelling to others |

**Table 3**

Comparison of regression algorithms

|  | Random Forest | | Support Vector Machine | | Linear Regression | |
| --- | --- | --- | --- | --- | --- | --- |
|  | *test* | *training* | *test* | *training* | *test* | *training* |
| MAE | 0.284 | 0.207 | 0.300 | 0.187 | 0.432 | 0.316 |
| MSE | **0.081** | **0.043** | 0.09 | 0.035 | 0.187 | 0.100 |

1. A list of examined indicators is provided in Table S1. [↑](#footnote-ref-1)
